# Structural insights into the Middle East respiratory syndrome coronavirus 4a protein and its dsRNA binding mechanism

**DOI:** 10.1038/s41598-017-11736-6

**Published:** 2017-09-12

**Authors:** Maria Batool, Masaud Shah, Mahesh Chandra Patra, Dhanusha Yesudhas, Sangdun Choi

**Affiliations:** Department of Molecular Science and Technology, Ajou University, Suwon, 16499 South Korea

## Abstract

Middle East respiratory syndrome coronavirus (MERS-CoV) has evolved to navigate through the sophisticated network of a host’s immune system. The immune evasion mechanism including type 1 interferon and protein kinase R-mediated antiviral stress responses has been recently attributed to the involvement of MERS-CoV protein 4a (p4a) that masks the viral dsRNA. However, the structural mechanism of how p4a recognizes and establishes contacts with dsRNA is not well explained. In this study, we report a dynamic mechanism deployed by p4a to engage the viral dsRNA and make it unavailable to the host immune system. Multiple variants of p4a-dsRNA were created and investigated through extensive molecular dynamics procedures to highlight crucial interfacial residues that may be used as potential pharmacophores for future drug development. The structural analysis revealed that p4a exhibits a typical αβββα fold structure, as found in other dsRNA-binding proteins. The α1 helix and the β1-β2 loop play a crucial role in recognizing and establishing contacts with the minor grooves of dsRNA. Further, mutational and binding free energy analyses suggested that in addition to K63 and K67, two other residues, K27 and W45, might also be crucial for p4a-dsRNA stability.

## Introduction

The innate immunity of host cells is the first line of defense that initiates a protective response against pathogenic microorganisms and viruses^1^. Germline-encoded receptors of the innate immune system protect the host cells from infection by pathogenic intruders^2^, while pathogens constantly evolve various strategies to circumvent the host’s protective responses^3^. Host cells are equipped with counter mechanisms to detect virus-encoded molecular patterns and propagate an antiviral response. Viral double-stranded RNA (dsRNA) is a well-characterized pathogen-associated molecular pattern recognized by cytosolic pattern recognition receptors retinoic acid inducible gene-1 (RIG-1), melanoma differentiation-associated protein 5 (MDA5), and endosomal toll-like receptor 3 (TLR3), resulting in type 1 interferon (IFN1) production^4^. Viruses employ a unique evasion mechanism by synthesizing proteins that hinder the IFN1 production and secretion pathways. For instance, influenza A virus uses non-structural protein 1 to bind dsRNA^5^, inhibiting RIG-1-like receptors and TLR3-dependent IFN1 synthesis. Dengue virus, on the other hand, prevents IRF3 phosphorylation through the non-structural protein 2B3 protease complex^6^. Similarly, Middle East respiratory syndrome coronavirus (MERS-CoV) implements a mechanism to evade dsRNA sensors including RIG-1, MDA5, and endosomal TLR3 of the host immune system. Subsequent studies have found that MERS-CoV is much more sensitive to IFN1 treatment than severe acute respiratory syndrome coronavirus (SARS-CoV)^7–10^. This viral interference in the host innate immune pathway enhances virus-induced disease progression and elevates the mortality rate to 60%^11, 12^.

MERS-CoV is a major cause of chronic respiratory diseases and the first case was reported in Jeddah in 2012^13^. The natural habitat of the virus is not known; however, phylogenetic analyses show that bat coronaviruses bCoV-HKU4 and bCoV-HKU5 are the closest neighbors to MERS-CoV, suggesting that the virus can be spread by bats^14^. The genome organization of MERS-CoV is similar to SARS-CoV, in which non-structural proteins responsible for genome replication cover two-thirds of the genome. The remaining parts of the genome encode structural (membrane, spike, nucleocapsid, and envelope proteins) and accessory proteins^15, 16^. Once MERS-CoV enters the cells with the help of dipeptidyl peptidase–4 receptors^17^, it starts replicating by manipulating and modulating the host cells’ metabolism^18^, including antigen presentation, apoptosis, mitogen-activated protein kinase, and innate immune response. MERS-CoV interrupts IFN1 response of the cell and may encode certain proteins that help the virus to escape from the host immune system. The accessory proteins 3, 4a, 4b, 5, and 8b, encoded by various open reading frames, inhibit IFN1 production in the infected cells, mainly in dendritic cells^19^.

Viral-dsRNA is the principal agent that triggers the host immune response mediated by RIG-1, MDA5^20, 21^, and endosomal TLR3. RIG-1 recognizes dsRNA through its dsRNA-binding domain (dsRBD) and undergoes conformational changes to expose its caspase activation and recruitment domain. Activated RIG-1 initiates a downstream signaling cascade involving mitochondrial antiviral signaling adaptor protein and kinases TBK1 and IKKε, leading to the activation and nuclear translocation of transcription factors IRF3 and IRF7, which facilitate IFN1 promoter activity^22^.

MERS-CoV utilizes protein 4a (p4a) to ensure its replication without being detected by the RIG-1 and MDA5 receptors^23^. Further, p4a contains a dsRNA-binding motif (DSRM), which binds directly to the viral dsRNA and masks its recognition by TLR3 and RIG-1^23^. Mutational studies have shown that two amino acids, K63 and K67, in the p4a-DRSM play crucial role in RNA association and p4a-dsRNA complex stability^22^. Other studies also provided consistent results that p4a antagonizes IFN1 induction in host cells^23^. Thus, the inhibition of p4a would allow the host cells to restore anti-MERS-CoV immune response.

Several research groups have been trying to formulate an effective anti-MERS-CoV vaccine or drug to prevent future epidemics^24–29^. Recently, a few drugs have been suggested to inhibit viral replication; however, these have not been tested *in vivo* ^30–33^. In this study, we conducted computational studies and constructed a three-dimensional (3D) model of p4a to understand the structural aspects and its dsRNA recognition mechanism. Based on the experimental knowledge learned and our analyses, we created multiple variants of p4a through *in silico* mutagenesis and vetted them for their underlying effects on p4a-dsRNA binding stability. The results from this study may be useful as a guide for future studies on developing high-affinity p4a inhibitors through rational drug design approaches.

## Results

### Protein modeling and p4a-dsRNA interface analyses

**Figure 1:**
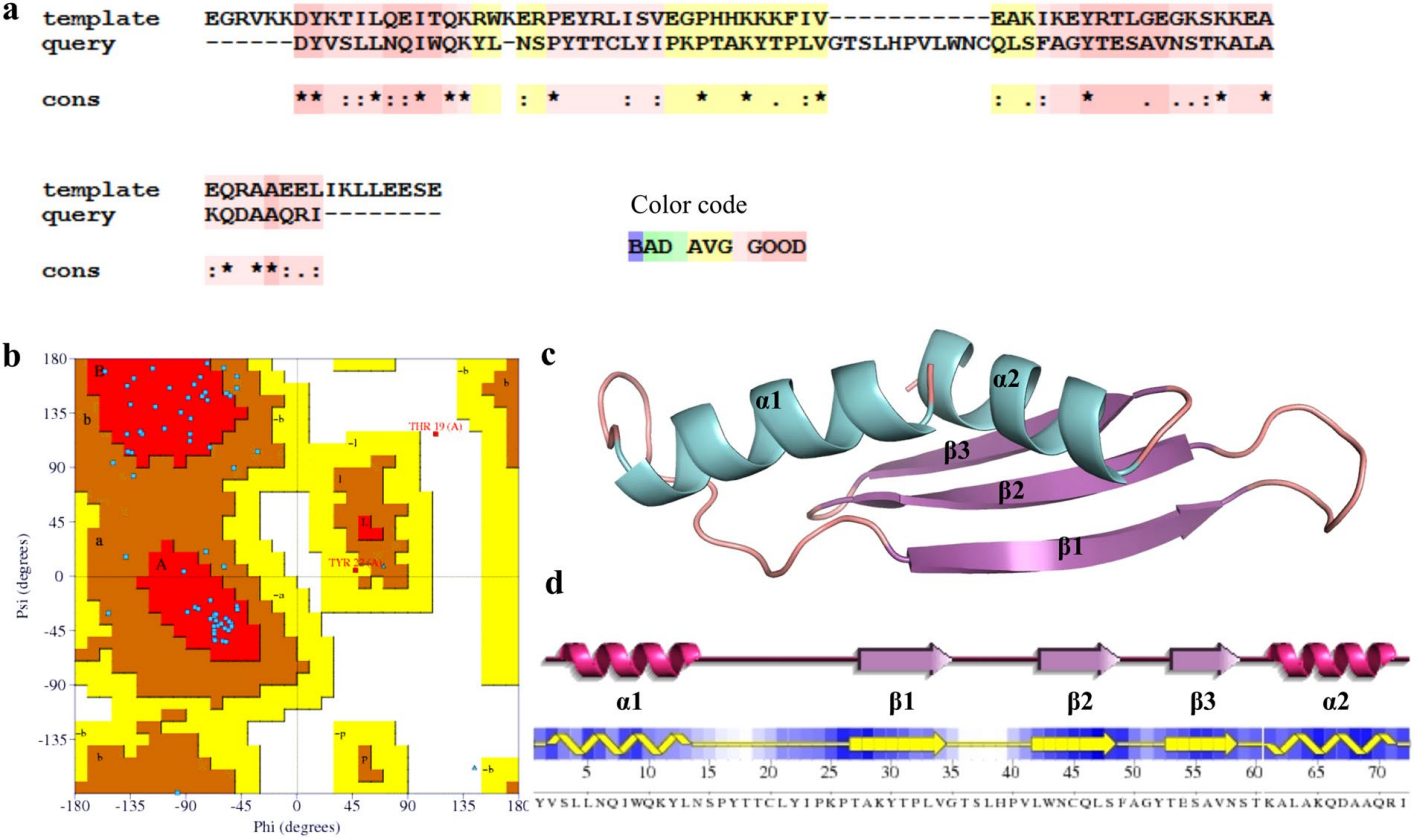
Structural representation of the MERS-CoV protein 4a. (**a**) Alignment of MERS-CoV p4a and template sequences. The quality of the alignment is highlighted using different colors. The rose color represents good alignment and the yellow color highlights average alignment between p4a and template residues. (**b**) A Ramachandran plot, depicting backbone dihedral angles ψ against φ of amino acid residues in a 3D model of p4a. The red color in the X-Y plot represents the core region, while the brown color represents the generously allowed region. (**c**) The 3D structure of the dsRNA-binding domain (dsRBD) of MERS-CoV p4a adopts a distinctive topology (αβββα) found in other DSRM-containing proteins. (**d**) The predicted secondary structure of p4a was in agreement with the built 3D model.

The high-resolution 3D structure of a protein and its interacting partners are of crucial concern when their interaction mechanisms need to be deciphered. In addition, only 3D atomic level structures can suggest plausible druggability of viral proteins. However, in this particular case, a high-resolution 3D structure of MERS-CoV p4a, alone or bound to dsRNA, is not available. Thus, p4a-DSRM, containing 72 amino acid residues, was modeled and investigated for its structural stability. 1YYK^34^, the crystal structure of *Aquifex aeolicus* dsRNA binding protein, was used as a template, with a TM-score of 0.7577 suggested by Iterative Threading ASSEmbly Refinement (I-TASSER) modeling server, to build p4a model. The sequence alignment of p4a with 1YYK shows a satisfactory normalized Z score of 1.27 (a normalized Z score > 1 is considered as a good score). The pairwise sequence alignment obtained from the T-COFFEE server also showed a satisfactory score of 76 (Fig. 1a). After the validation of structures obtained from different servers and tools (Supplementary Table S1), the 3D structure obtained from I-TASSER was selected as the best model for further studies. The 3D structure validation and protein geometry results were found to be satisfactory, as provided by the ProSA web server. Thr19, Tyr22, and Ala50, located in loop regions, were found to be outside of the core region in the Ramachandran plot (Fig. 1b). However, an overall ERRAT quality factor and Z-score values, calculated using ProSA-web, were found to be satisfactory (Supplementary Table S1). The obtained structure of p4a-DSRM exhibited a typical dsRBD structure that adopts an αβββα fold (Fig. 1c). This particular folded structure has been endowed with the ability to recognize and bind to dsRNA^35^. To validate the arrangement of the secondary structural elements, the modeled protein was compared with the predicted secondary structure. MERS-CoV p4a consists of 33% helix, 33% strands, and 34% coils. In the model, the helices α1 and α2 span from residue positions 3 to 13 and 61 to 71, while β1, β2, and β3 span from residues 27 to 34, 42 to 48, and 53 to 58, respectively (Fig. 1d).

The optimized p4a model was selected and subjected to a structural similarity search. The TM-align^36^ web server was used to match the structural feature of the query model to all Protein Data Bank (PDB) structures. Interestingly, we found that all of the top ten structures having the highest structural similarity with p4a were dsRBD-containing proteins (Table 1). Based on the TM-score and high similarity in protein structures, as predicted through a global structure comparison, it is very likely that the target protein has the same function as the query. For a plausible functional annotation of p4a, a meta-server COACH^37^ was used. COACH also suggested the same top two structures suggested by TM-align.

**Table 1:** Proteins structurally and functionally close to MERS-CoV p4a.

Rank	PDB	TM-s	RMSD^1^	IDEN^2^	Cov^3^
1	1di2A	0.81	0.77	0.161	0.861
2	3adlA	0.803	0.84	0.194	0.861
3	2ez6B	0.793	1.09	0.21	0.861
4	1uhzA	0.78	1.22	0.21	0.861
5	1rc7A2	0.78	1.18	0.21	0.861
6	2cpnA	0.727	1.38	0.21	0.861
7	3adgA	0.723	1.39	0.246	0.847
8	3llhA	0.72	0.91	0.161	0.778
9	1x49A	0.718	1.4	0.217	0.833
10	4dkkA	0.717	1.35	0.103	0.806

Ranking of similar proteins is based on the TM-score of the structural alignment between the query structure and known structures in the PDB library.

^1^RMSD is the RMSD between residues that are structurally aligned by TM-align.

^2^IDEN is the percentage sequence identity in the structurally aligned region.

^3^Cov represents the coverage of the alignment by TM-align and is equal to the number of structurally aligned residues divided by the length of the query protein.

Knowing the structural features and plausible dsRNA binding residues, we further investigated the RNA interaction mechanism of MERS-CoV p4a. A protein-RNA docking protocol was used to identify the binding interface and crucial residues in p4a. Residues in the α1 helix and β1-β2 loop regions make contact with the minor grooves of the dsRNA. However, K63 and K67, present in the α2 helix, make dominant contacts with the major groove of RNA (Supplementary Fig. S1). To decipher the involvement of all interfacial residues in p4a-dsRNA interaction and stability, we extended our analysis to computational mutagenesis.

### Computational mutageneses of p4a-dsRNA interfacial amino acids and their subsequent impact

An *in silico* mutagenesis study was performed to identify the specific residues of p4a influencing dsRNA binding. The binding free energy difference between a given residue and an alanine substitution suggests the significance of that particular residue in complex stability. The involvement of a particular amino acid, in terms of energy, in the p4a-dsRNA complex can be calculated in the form of relative binding affinity (dAffinity) and relative thermostability (dStability) by the protein design tool of molecular operating environment (MOE) program (Table 2). Positive dAffinity values indicate that the RNA-binding affinity of p4a is reduced if a particular residue is mutated to alanine and vice versa. Similarly, mutants with positive dStability scores show less stable mutations. This suggests that native-to-alanine mutants with positive and high dAffinity and dStability values are hotspots and play a vital role in p4a-dsRNA complex formation (Supplementary Table S2). To validate this, we used the I-Mutant 2.0 web server, which generated similar results (Table 2). The residues were labeled as hot, warm, and null based on the free energy value differences^38^. Residues with a change in free energy value (ΔΔG) less than 0.5 kcal/mol were assigned as null or unimportant residues. Those having values between 0.5 and 1.5 kcal/mol were assigned as warm residues and those with values more than 1.5 kcal/mol were assigned as hotspot residues. ΔΔG^binding^ represents the difference between the binding energy of p4a mutants and wild type, at a particular position.

$${{\rm{\Delta }}{\rm{\Delta }}{\rm{G}}}^{{\rm{binding}}}={\rm{\Delta }}{\rm{G}}\,{{\rm{binding}}}^{{\rm{MUT}}}-\,{\rm{\Delta }}{\rm{G}}\,{{\rm{binding}}}^{{\rm{WT}}}$$

**Table 2:** Alanine scanning mutagenesis.

Mutation	ΔΔG^1^ (kcal/mol)	dAffinity^2^	dStability^3^
N8A	−1.25	3.35	0.523
K27A	−1.18	10.11	0.14
W45A	−2.90	5.61	5.60
K63A	−0.14	0.22	−0.70
K67A	0.11	0.57	−0.20

Mutational hotspot residues generated by the MOE program and I-Mutant server. ΔΔG^binding^ is the difference between the binding energy of a mutant (ΔG^MUT^) and the wild type (ΔG^WT^). A positive dAffinity value indicates reduced binding affinity of the complex and positive dStability means less stable mutations.

^1^Difference in binding energies of mutant and wild type residue obtained from I-Mutant server.

^2^The relative binding affinity of the mutation to the wild type protein. A more negative value indicates a mutation with better affinity.

^3^The relative thermostability of the mutation with respect to the wild type protein. A more negative value indicates a more stable mutation.

Our results showed that among the ten interfacial residues selected for mutation, two previously reported substitutions, K63A and K67A, were found to be comparatively less important in terms of change in stability and affinity as compared to N8, K27, and W45 (Table 2). However, K63 and K67 were among the top five hotspots. Three other residues N8, K27, and W45 were considered as hotspots and their ΔΔG were logged as −1.25, −1.18, and −2.90 (kcal/mol), respectively, whereas their dStability values were 3.35, 0.14, and 5.61 (kcal/mol), respectively (Table 2). These results imply that W45 located in the β2 strand of p4a may have a significant impact on and could disrupt p4a-dsRNA interaction. However, the selected hotspots were further investigated by molecular dynamic (MD) simulations to highlight their direct or indirect impact on p4a-dsRNA stability.

### Structural dynamics of the hotspot residues and their impact on p4a-dsRNA stability

**Figure 2:**
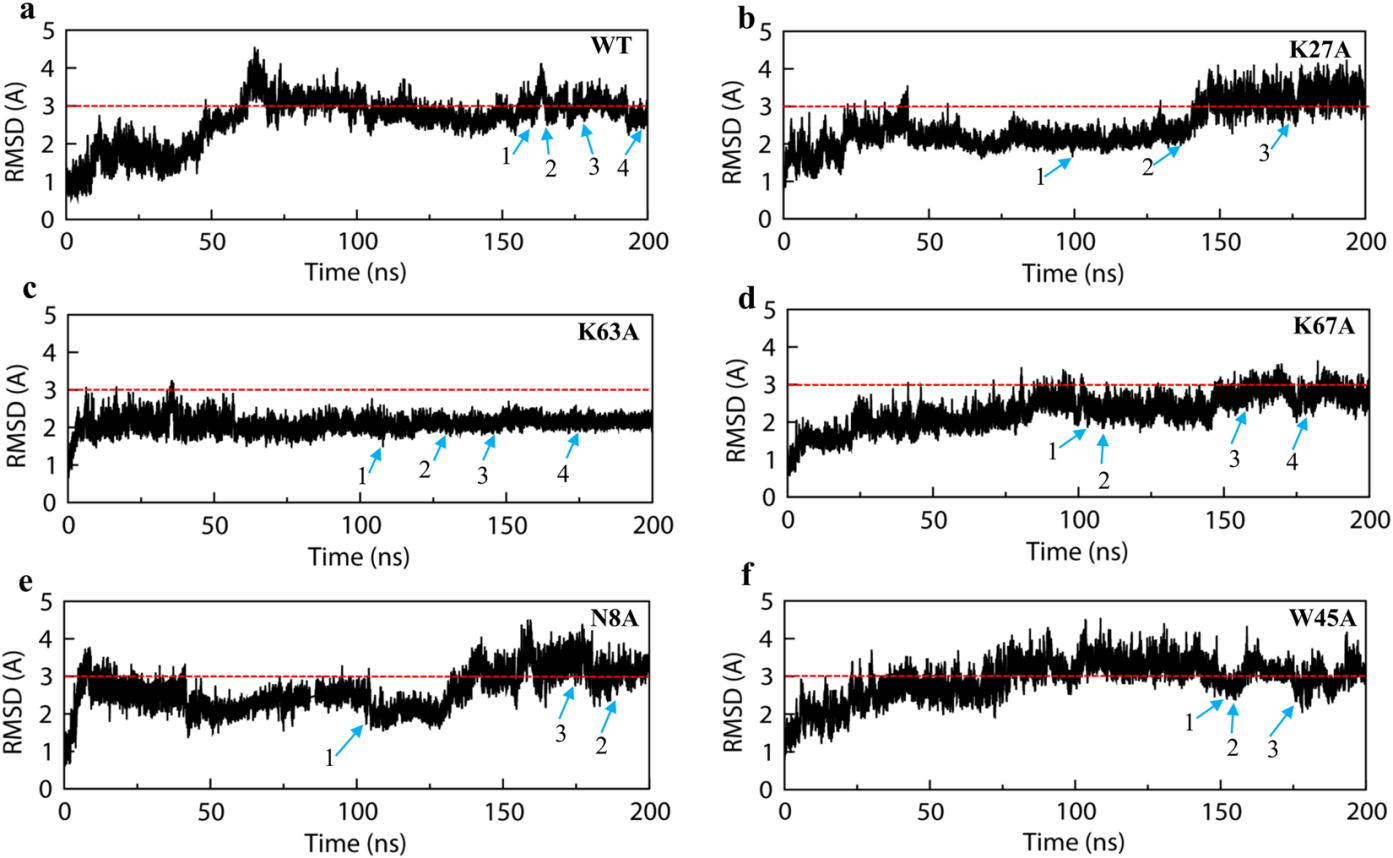
Root mean square deviation (RMSD) of the wild type and alanine mutants of p4a. The RMSD graph shows the deviation of the backbone atoms (from the initial state) of the RNA-binding domain of p4a over the 200 ns simulation (**a**) wild type, (**b**) K27A, (**c**) K63A, (**d**) K67A, (**e**) N8A, and (**f**) W45A.

The wild type and mutant complexes were simulated three times in an explicit water environment for 200 ns each. The deviation of backbone atoms was examined by the root mean square deviation (RMSD). We observed that the RMSD of the wild type complex substantially increased in the beginning, up to 4 Å around 60 ns. However, this deviation then reduced gradually and oscillated around 3 Å after 60 ns MD simulations (Fig. 2a). In contrast, the mutant complexes showed dissimilar patterns of backbone deviation. The backbone RMSD of all complexes exhibited variable behavior during the 200 ns MD simulations (Fig. 2b–f). The K63A mutant had a stable RMSD (~2 Å) throughout the simulation and reached equilibrium in the end (Fig. 2c), whereas the N8A and W45A mutants exhibited high RMSD fluctuations throughout the MD run (Fig. 2e and f). The variations in the backbone fluctuation of the mutant complexes point to the involvement of these residues in p4a-dsRNA stability. The superimposed RMSD graphs of all complexes of each repeat are provided in Supplementary Fig. S2.

**Figure 3:**
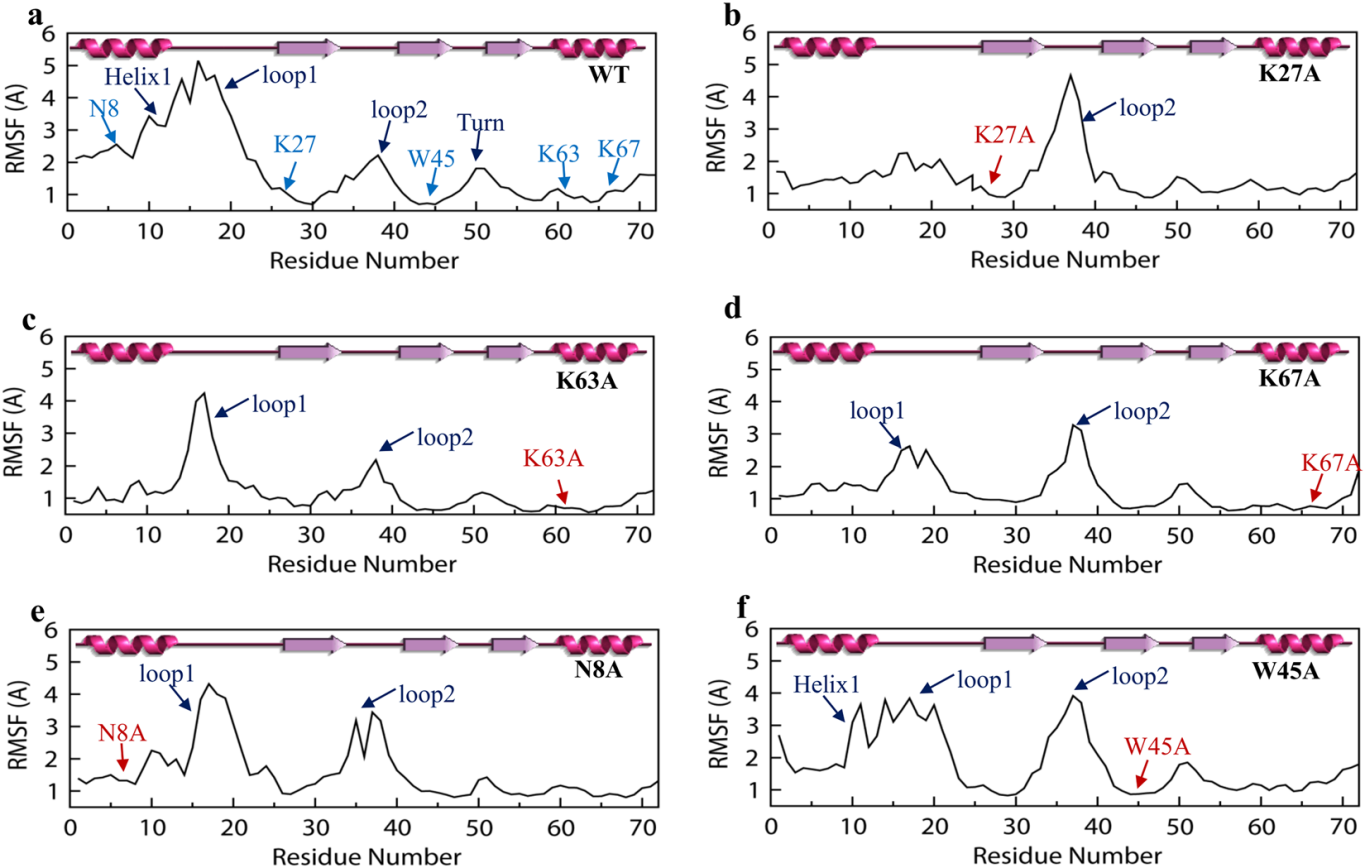
Root mean square fluctuation (RMSF) of the wild type and alanine mutants of MERS-CoV p4a. Residue fluctuations, monitored for the Cα atoms of the protein over the entire trajectory, are shown. Each panel represents variants of p4a and navy blue pointers highlight the most fluctuating elements and positions of mutants. Blue arrows in (**a**) highlight the residues in the wild type before mutation and red arrows indicate the mutant residue in (**b**) K27A, (**c**) K63A, (**d**) K67A, (**e**) N8A, and (**f**) W45A.

To understand the effect of individual amino acids of p4a-dsRNA wild type and mutant complexes, we analyzed the root mean square fluctuations (RMSF). The wild type complex showed greater fluctuations between residues 10 and 20 (part of the α1 helix region), while the rest of the residues were quite steady during the MD simulation (Fig. 3a). However, the mutant complexes displayed local fluctuations at various positions (Fig. 3b–f). A closer analysis revealed that all p4a-mutants (except K27A) had distinct RMSF peaks between residues 10 and 20 and they contained few additional peaks that did not occur in the wild type complex. Therefore, the stability of specific residues during the MD simulation might be dependent on their interactions with the original residues that were mutated to alanine. Interestingly, the substitutions N8A, K27A, W45A, and K67A resulted in an increased fluctuation between residues 30 and 40 (the β1-β2 loop region), while K63A was relatively stable at this position (Fig. 3b–f), which is consistent with the RMSD observation. This β1-β2 loop plays a crucial role in identifying the minor groove in dsRNA. In N8A (Fig. 3e), the loop residues spanning from 13 to 26 and 31 to 42 showed larger RMSF peaks, whereas, in W45A (Fig. 3f), the α1 helix residues spanning from 3 to 12 displayed high fluctuations. These findings suggest that mutating these residues might not have any direct effect on p4a-dsRNA binding; however, they may distantly affect the stability of other residues that are vital in the interaction.

After observing the backbone deviations and local fluctuations of the complexes, we calculated the radius of gyration (Rg) to understand the overall compactness of the wild type and mutant p4a complexes. All of the mutant complexes showed discrete patterns for Rg values throughout the MD simulation. In particular, the mutants K27A, W45A, and K63A were distinct as compared to the wild type in terms of Rg values, likely due to the point mutations (Supplementary Fig. S3). To investigate the correlated residue motions, contact maps were plotted as a function of residue number. The residue contact maps showed a few structural deviations from positions 20 to 30 and 50 to 60 in all mutants as compared to the wild type. However, in W45A, these internal structural changes were found to be minor (Supplementary Fig. S4).

### Intermolecular interactions between dsRNA and p4a variants

**Figure 4:**
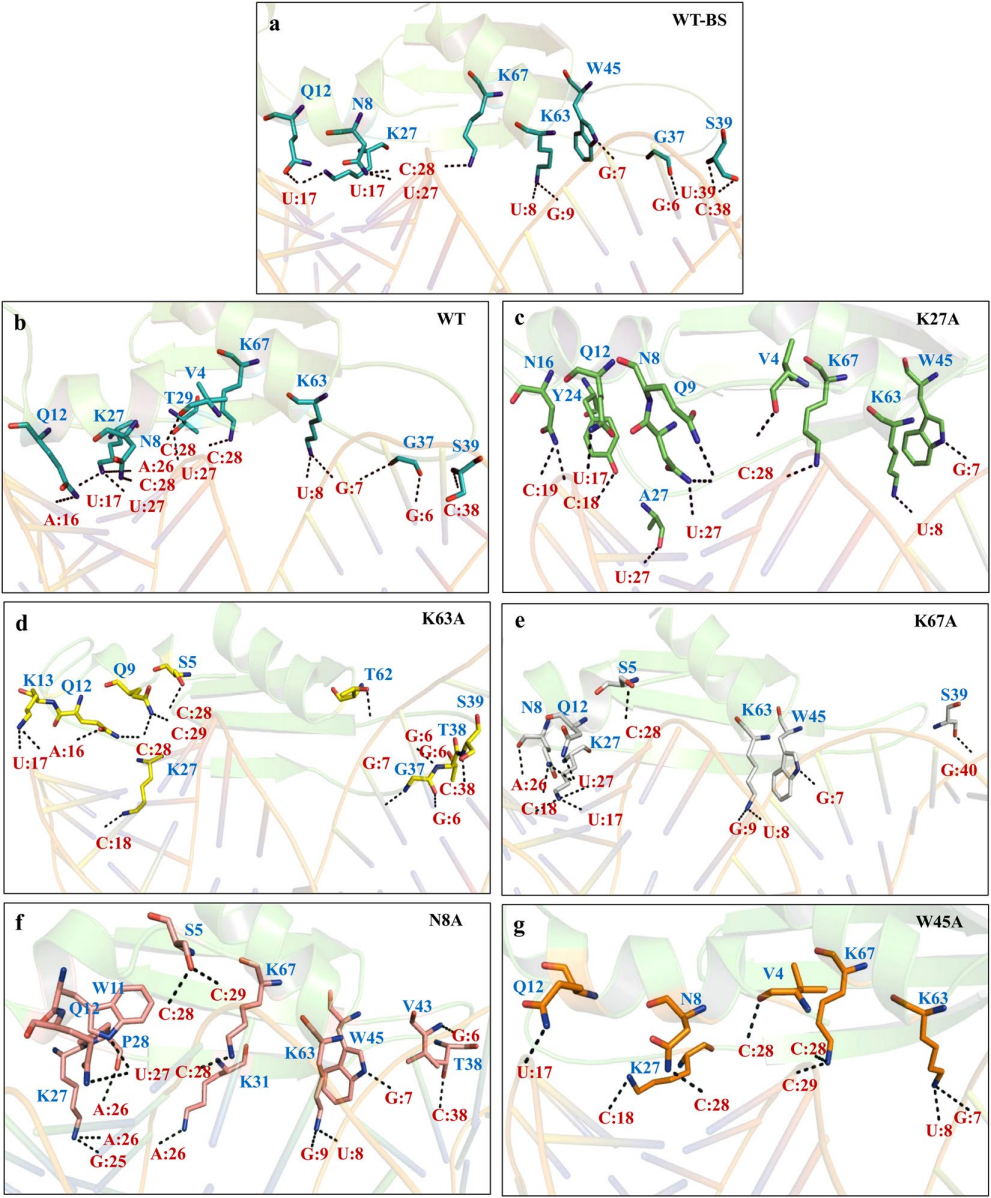
Hydrogen bonds calculated between the wild type and mutant p4a-dsRNA complexes before and after simulations. Changes in the number of hydrogen bonds were calculated for the wild type and mutant p4a-dsRNA complexes before and after the 200 ns MD simulations. (**a**) The type of residues and nucleotides involved in h-bonding are shown for the wild type p4a before simulation, while the change in the number and type of residues involved in h-bonding after the MD simulation are shown as follows: (**b**) wild type, (**c**) K27A, (**d**) K63A, (**e**) K67A, (**f**) N8A, and (**g**) W45A. Blue and red colors show the amino acids and RNA bases, respectively.

The MD simulations were used to optimize and understand the possible binding mechanism of dsRNA with p4a as well as its alanine-variants. In order to understand the effect of alanine mutations on the interaction of p4a with dsRNA, we performed a comparative analysis of intermolecular hydrogen bond (h-bond**)** patterns before and after the 200 ns MD simulations (Fig. 4). Analysis of h-bonds formed between dsRNA and wild type p4a revealed that the hotspot residues K27, K63, K67, N8, and W45 make h-bonds with dsRNA. Time-dependent calculations of interaction distances revealed that K67, K63, K27, and W45 had consistent interactions with dsRNA during the MD simulations (Supplementary Fig. S5a). Mutating these residues substantially affected the intermolecular h-bond numbers between p4a and dsRNA. The N8A and W45A variants surprisingly reduced this number from 12 (wild type) to ~7 (mutants) (Fig. 4f and g; Supplementary Fig. S5b). The K67A variant also exhibited a reduction trend in h-bond numbers, similar to that observed in the N8A and W45A variants. The decrease in the h-bond number in the mutants W45A and N8A highlight the importance of these residues in the binding of dsRNA and p4a. Both h-bond numbers and RMSF values indicate that mutations at these three positions have a distant effect on loop 2 located between β1 and β2 strands.

### Molecular dynamic motions of p4a variants

In order to identify the dominant motions in p4a-dsRNA wild type and mutant complexes, a principal component analysis (PCA) was performed, in which most of the combined dominant motions were captured by the first 10 eigenvectors. The amplitude of the corresponding eigenvalues gradually dropped and attained constrained and more localized fluctuations. The first 10 eigenvectors were generated from the trajectory and inspected for their contribution to the total fluctuation of p4a variants. PCA for wild type p4a indicated that the first five eigenvectors accounted for ~80% of the variance in the motion observed in the MD trajectory. A similar pattern was exhibited by the N8A, W45A, and K63A variants. However, in the case of K27A and K67A, the first eigenvector accounted for three-fourths of the total variance of the first five eigenvectors (Supplementary Fig. S6). The first three eigenvectors were plotted against each other and two-dimensional plots were generated to compare the conceivable attributed motions (Supplementary Fig. S6). These plots display the variance in the conformational distribution of the p4a-dsRNA complex indicated by each dot in the respective plot. The continuous color representation (from blue to white to red) highlights the periodic jumps between these conformations.

**Figure 5:**
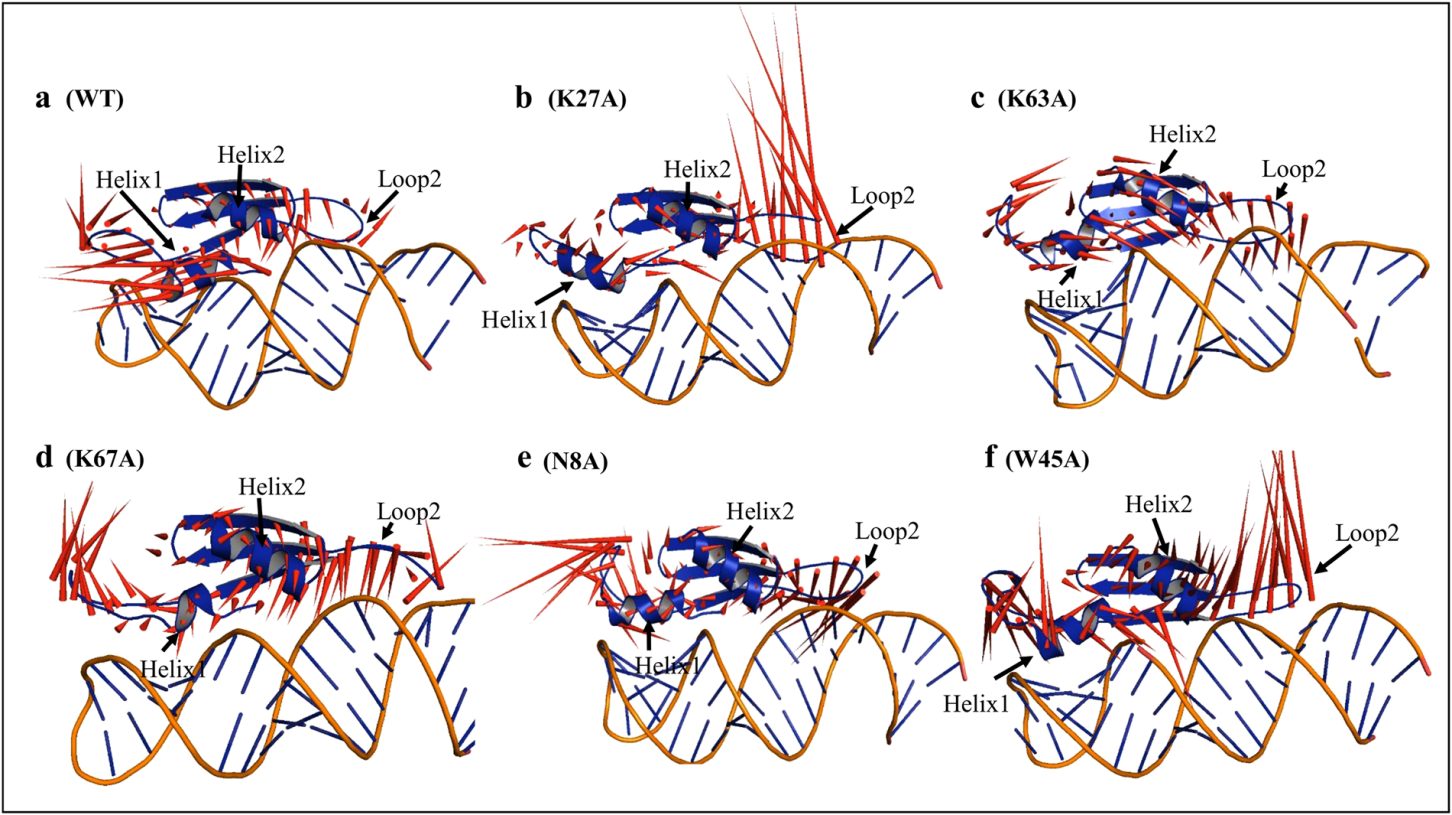
Principal component analysis (PCA) of p4a. Porcupine plots are drawn using PyMOL to visualize the movements of the first eigenvector obtained from the PCA analysis. In (**a**) wild type, (**b**) K27A, (**c**) K63A, (**d**) K67A, (**e**) N8A, and (**f**) W45A, red cones depict the direction of movements of protein subdomains and the length of a cone represents the magnitude of the movement.

Porcupine plots were constructed to visualize the movements graphically using the structural coordinates of the first eigenvector for each variant, as shown in Fig. 5. In the wild type, helix α1 had the highest motion parallel to the axis of the dsRNA; however, loop 2 exhibited a dormant behavior. As shown in Fig. 5b and f, substituting K27 and W45 with alanine greatly affected the stability of loop 2. The amplitude of the eigenvectors was substantially high in these two mutants, indicating a moving tendency away from the dsRNA (Fig. 5b, f, and Supplementary Fig. S7). Other mutants N8A and K67A also exhibited considerable fluctuations in loop 1; however, this loop was relatively less important for p4a-dsRNA stability, as we could not observe any considerable contribution to p4a-dsRNA interaction from this loop. Overall, the domain motions showed that the mutations in the β1 or β2 strands or the loop between these two strands could greatly influence the dynamics of dsRNA binding to p4a.

**Figure 6:**
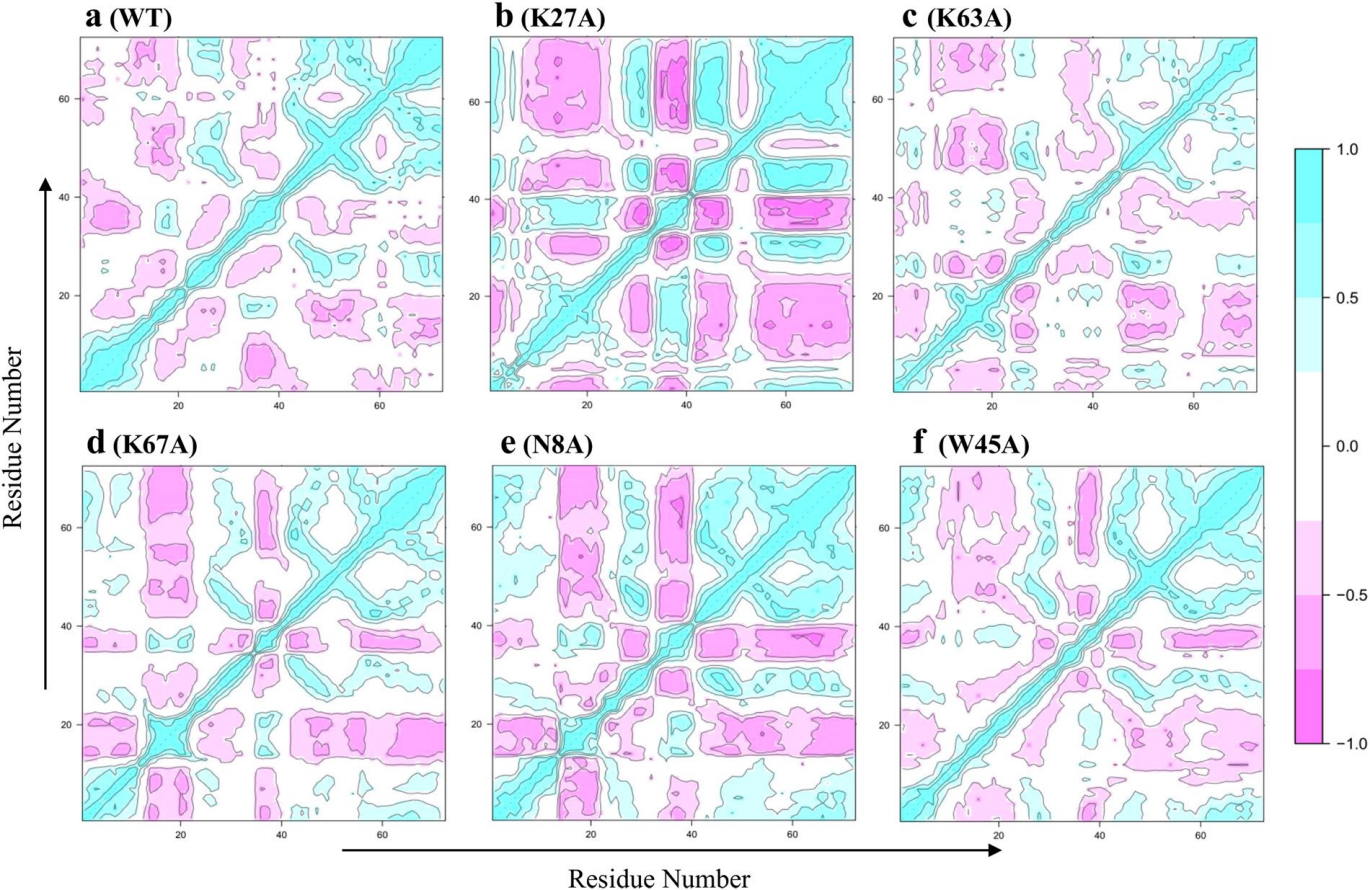
Dynamic cross correlation map (DCCM). The DCCM map for (**a**) wild type, (**b**) K27A, (**c**) K63A, (**d**) K67A, (**e**) N8A, and (**f**) W45A shows the correlated motions of protein residues in wild type and mutant complexes. The cyan color represents positive correlation and the pink color represents negative correlation. The color gradients represent a gradual decrease in the correlation.

To investigate the functional displacements of p4a atoms as a function of time, we constructed and analyzed a dynamics cross correlation matrix (DCCM). Different patterns of correlated motions were observed in all mutant complexes compared to wild type complexes. However, the difference in the correlation of atomic displacements was prominent in K27A, while K63A presented a smaller number of correlated motions. Wild type and K63A showed a similar behavior for correlated displacements, while N8A and K67A showed negatively correlated displacements (Fig. 6). W45A displayed weak, negatively correlated motions at residues 35 to 72; however, it showed partial correlations among the residues before 35. In the wild type complex, helix α1 of K67A and N8A had highly correlated movements. The movement of loop 2 varied among different complexes, which agrees with the corresponding RMSF (Fig. 3) and porcupine plot data (Fig. 5). In the wild type, the atomic displacement was not correlated initially, but later it indicated slightly negatively correlated motions as the simulation progressed. In all other complexes, loop 2 revealed negatively correlated movements except for a few residues having minor correlated displacements. Helix α2 depicted similar correlated movements in all complexes except for the wild type complex. In short, all mutants exhibited different correlated motions than did the wild type complex, where most of the residues showed negative correlations except for those of helix α2.

### Exploring the transition pathway from metastable to native states

**Figure 7:**
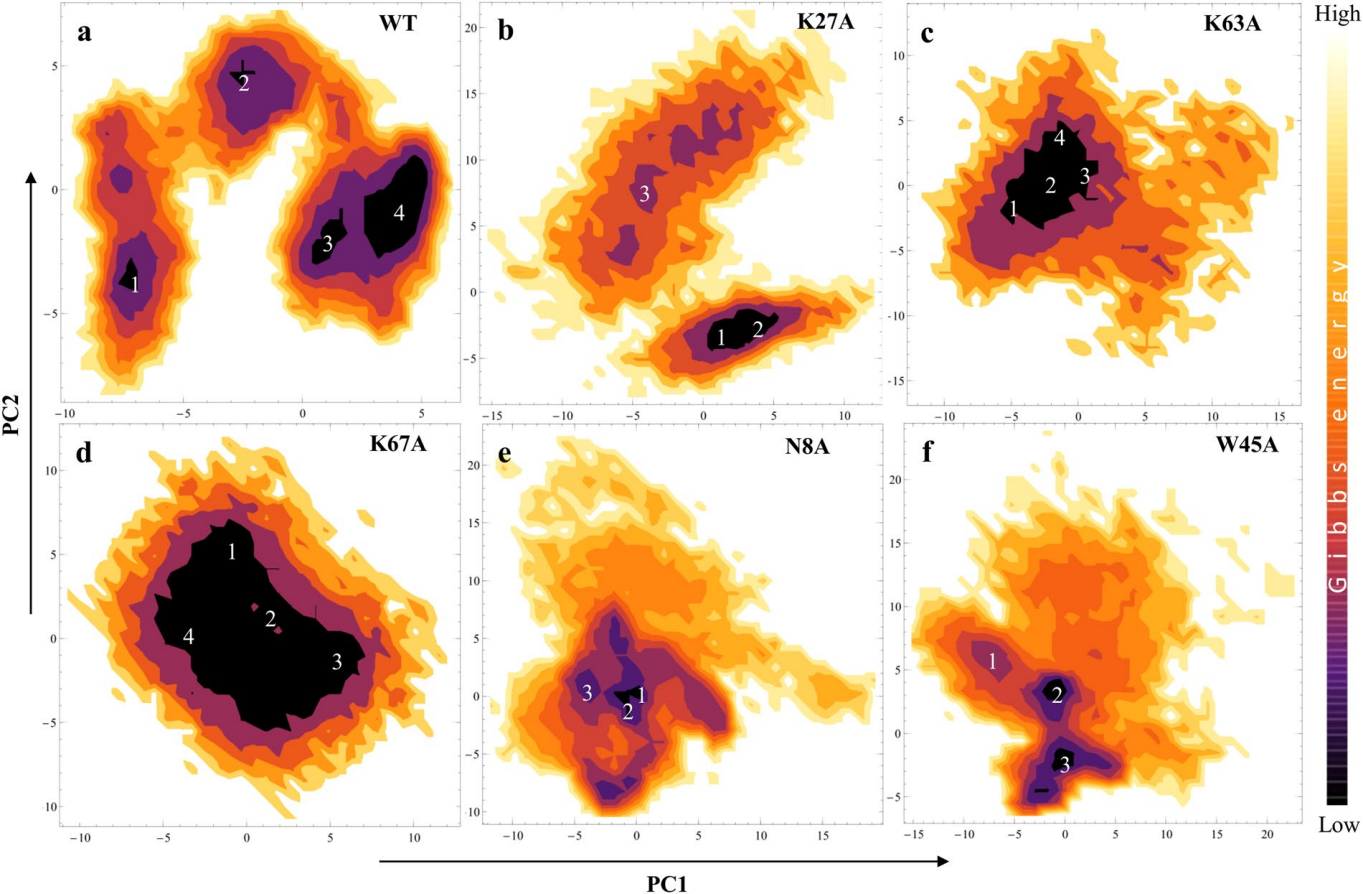
Free energy landscape (FEL) of the p4a and its variants. (**a**) Wild type, (**b**) K27A, (**c**) K63A, (**d**) K67A, (**e**) N8A, and (**f**) W45A represent the FEL obtained from the first two eigenvectors. The black color represents the lowest energy state while a continuous change from purple to yellow color highlights an increase in the Gibbs energy of the respective complexes. The numbers on each FEL plot correspond to the coordinates extracted from a specific time frame and analyzed for structural evolution.

The first two eigenvectors were used to plot and calculate the free energy landscape (FEL) and to determine the dominant native and metastable states of p4a and its variants during the 200 ns trajectory time. Structural coordinates were extracted from the low energy states to understand structural evolution. In Fig. 7, black color represents the lowest Gibbs energy states, while the numbers represent the positions of the structural coordinates sampled from that locus on the FEL plot. The occurrence time of these coordinates was then tracked and marked on the RMSD plots of the respective variants (Fig. 2). The FEL plot showed that the wild type attained three different energy states (two metastable and one native) separated by high-energy barriers; however, it remained in one energy state for most of the time (Fig. 7a). The experimentally reported mutant K63A remained in one dominant native state and did not show a transition to any other native or metastable state during the entire simulation (Fig. 7c). This was also recorded in the backbone RMSD plot, where K63A remained stable throughout its MD trajectory (Fig. 2c). The PDB coordinates, obtained from four different points at the lowest energy state of the FEL plot, corresponded to multiple points between 116–200 ns on the RMSD plot. Other variants, however, exhibited different behavior on the FEL plots and different Gibbs energies. N8A and K27A visited multiple metastable states during their structural evolution in MD simulations and were separated by low- and high-energy barriers, respectively (Fig. 7b, and e). The K27A variant, however, exhibited a clear transition from one state to another energy state. Interestingly, the same transition trend was observed in its backbone RMSD plot, where the K27A complex remained stable until ~130 ns of the MD run; its RMSD then suddenly increased and started fluctuating relatively high, as compared to the first 130 ns, for the last 50 ns (Fig. 2b). The W45A and N8A variants exhibited similar behavior; however, the W45A plot was less diffused and the states were separated by lower-energy barriers compared to N8A. The FEL map depicted that the second experimentally reported variant K67A stayed at one native state during the entire simulation; nonetheless, this energy state was more diffused and broader than the K63A variant (Fig. 7d).

### Monitoring RNA-binding affinity of p4a variants in terms of binding free energy

The energetic parameters that describe the binding interaction between dsRNA and p4a in both wild type and mutant complexes were analyzed by the Molecular Mechanics Poisson−Boltzmann Surface Area (MMPBSA) approach. For this purpose, we extracted 500 snapshots from the trajectory of the last 10 ns of the MD simulation. Partitioning of the binding free energy into its individual components; i.e. van der Waals (vdW), electrostatic, polar solvation, and solvent accessible surface area (SASA) energies, revealed important factors to analyze the affinity of the p4a wild type and its mutants with dsRNA (Table 3).

**Table 3:** MMPBSA binding free energy (kJ/mol) partitioning of wild type and mutant 4a-dsRNA complexes.

Mutants	^1^Δ_vdW_	^2^Δ_elec_	^3^Δ_ps_	^4^Δ_sasa_	^5^ΔG_tot_
Wild type	−503.7 (24.6)	−7359.1 (153.8)	1819.7 (144.8)	−49.2 (2.3)	−6092.3 (155.8)
K27A	−305.6 (30.1)	−7212.5 (200.0)	1681.5 (184.8)	−34.4 (2.8)	−5871.0 (103.7)
K63A	−459.5 (26.1)	−5547.1 (143.4)	1508.3 (123.8)	−46.5 (2.1)	−4544.9 (139.3)
K67A	−406.8 (24.8)	−5860.4 (108.5)	1555.2 (75.1)	−40.9 (2.4)	−4753.0 (104.3)
N8A	−373.2 (27.4)	−6875.2 (135.8)	1280.5 (100.5)	−38.9 (2.5)	−6006.8 (121.7)
W45A	−373.8 (43.5)	−7365.1 (138.3)	1828.1 (104.7)	−41.2 (4.8)	−5952.0 (132.0)

^1^Van der Waals energy, ^2^Electrostatic energy, ^3^Polar solvation energy, ^4^Solvent accessible surface area energy, ^5^Total binding energy, Standard errors are shown in brackets.

The wild type complex showed the highest cumulative binding energy (−6,092.3 kJ/mol), contributed to by high individual energy terms as compared to the mutant complexes. Interestingly, the total binding energy (−5,952.0 kJ/mol), including electrostatic energy (−7,365.1 kJ/mol) and polar solvation energy (1,828.1 kJ/mol), of W45A was very close to the wild type complex. However, the vdW energy was considerably increased (−373.8 (43.5) kJ/mol). This is because the hydrophobic indole sidechain of W45 establishes two contacts (one hydrogen bond donor and one π-C) with ribose sugars of the RNA. W45A has less influence on the total electrostatic state of the p4a-dsRNA complex but considerably affects the vdW energy. This might be the reason why N8A and W45A showed insignificant changes in binding free energy but considerably influenced the overall stability of the complexes (Table 2).

Among the mutant complexes, K63A and K67A showed the lowest cumulative binding energies (−4,544.9 and −4,753.0 kJ/mol, respectively), suggesting that these positions could be essential for binding dsRNA predominantly through electrostatic interactions, given that positively charged K63 and K67 are complementary to the negatively charged dsRNA. However, this decreasing trend in the binding affinity was not witnessed for the K27A mutant, although comparatively lower total binding energy (5,871.0 kJ/mol), vdW energy (−305.6 kJ/mol), and SASA energy (−34.4 kJ/mol) scores were recorded than for the wild type. In all complexes, a positive polar solvation energy opposed complex formation, but strong electrostatic, vdW, and SASA energies favored complex formation. Taken together, the order of dsRNA binding affinities could be summarized as wild type > N8A > W45A > K27A > K67A > K63A. The positively charged residues K27, K63, and K67 on the surface seem to be highly crucial for dsRNA recognition. These sites could be further investigated through site-directed mutagenesis to unveil their cumulative and individual roles in dsRNA recognition that helps the virus to escape the immune response. Small molecules or peptides having active electrostatic surfaces could possibly hinder the interaction between p4a and dsRNA and restore the cellular antiviral response.

## Discussion

We adopted an extensive computational procedure to unveil the molecular mechanisms for recognition of dsRNA by p4a and establishment of interactions with dsRNA to bypass the host antiviral response. As mentioned earlier, p4a contains a dsRBD^23^. Our protein model also suggested that p4a exhibited a well-folded structure acquiring a typical αβββα pattern (Fig. 1c) and docking analysis showed that p4a recognizes dsRNA in a shape-specific manner by establishing contacts with its major and minor grooves. Similar results have also been reported previously, where adenosine deaminase RNA specific 2 (ADAR2) recognizes stem loop mRNA^39^. ADAR2 is an RNA transcript-editing enzyme that uses a reaction known as hydrolytic deamination. Previous studies reported that two conserved lysine residues at positions 63 and 67 are vital for the p4a-dsRNA interaction. Mutating these residues render p4a incapable of blocking IFN production in MERS-CoV-infected cells^22^. Interestingly, we found that these residues retained their contacts with dsRNA after subjecting the p4a-dsRNA wild type complex to 200 ns MD simulations. These preliminary findings support the results of previous studies and further suggest that other amino acid residues play a crucial role in p4a-dsRNA interaction and could be helpful in blocking p4a activity. To examine the role of each residue present at the interface of p4a and dsRNA, computational alanine scanning was used. In addition to the previously reported residues, K63 and K67, we found three more residues, N8, K27, and W45, that could disrupt the p4a-dsRNA interaction. A rigorous computational analysis was performed to pinpoint the role of these newly identified hotspot residues, using MD simulations and free energy calculations. An overall trajectory analysis through RMSD, RMSF, and essential dynamics revealed that the newly created variants displayed few variations in the 3D structure that might affect their affinity toward dsRNA. Interestingly, of the two experimentally reported variants, K63A showed a lower structural instability than did K67A when compared to the others (Figs 2 and 4). This indicates that the loss of the dsRNA binding strength in this mutant may require a different explanation. However, K67A showed marked deviations in RMSD, RMSF, and domain motions. All of the newly constructed alanine variants demonstrated a discrete pattern of structural dynamics, which is interesting because all of them had the same starting 3D configurations except for a single residue substitution. This is because the substitution of a single amino acid with alanine is sufficient to cause differential protein dynamics affecting ligand-binding capability in simulated or natural conditions.

The binding free energy calculations revealed that p4a had a decreased affinity toward dsRNA in K67A, K63A, and K27A mutants, while W45A and N8A possessed similar affinity to the wild type. This implies that among the newly identified computational point mutations, K27 might play a similar role to the other positively charged experimental mutations, K63A and K67A. The reduced affinity of K27A may be attributed to its positively charged side chain that provides increased stability to the negatively charged dsRNA by means of strong electrostatic interactions. The substitution of lysine at position 27 with alanine disrupts the binding affinity of K27A and dsRNA. However, the structural instability in p4a caused by W45A and N8 substitutions cannot be overlooked.

Hydrogen bonding in p4a-dsRNA wild type complex is strong. The strength of the p4a-dsRNA interaction was lost in the W45A mutant, which showed that residue W45 might play an important role in the binding of p4a to dsRNA. Further computational analysis showed that mutants W45A and K27A, other than the experimentally reported mutants, led to an excess loss of binding affinity. In this work, MD simulations were performed to understand the dsRNA-p4a binding mechanism and to reveal the important residues, which play a key role in binding. The wild type and mutant structures were stable during the 200 ns of the simulations but the average RMSD of mutant W45A was above 3 Å. Similarly, in W45A, the number of h-bonds constantly decreased with the MD trajectory over 200 ns. A significant decrease in the binding affinity calculations suggests that W45A and K27A are important for dsRNA and p4a binding, which can be targeted to block their interaction. These structural insights suggest that MERS-CoV p4a acquires a defined DSRM fold and recognizes dsRNA in a shape-dependent manner. The α1 helix and loop 2 are crucial for dsRNA recognition and establishing contacts with the minor grooves of dsRNA. However, fluctuation of loop 2 in the variants during MD simulations indicated a loss of binding strength between p4a and dsRNA.

The overall surface analysis suggested that p4a did not possess any defined ligand-binding hydrophobic pocket, which could make this protein an unpromising drug target. However, understanding of the surface electrostatics can lead to the design of electrostatically active small molecules that could hinder p4a-dsRNA interaction.

## Methods

### Molecular Modeling

The importance of p4a in viral pathogenesis and host immune evasion has been extensively investigated through *in vitro* assays; however, no structural studies have been conducted to unveil its RNA binding mechanism. MERS-CoV p4a is 109 amino acids long and contains an approximately 70–72 amino acid-long DSRM. To model the p4a DSRM, a template structure was searched against a PDB^40, 41^ by BLAST and through the threading-based modeling server, I-TASSER. The template structures selected by BLAST and the I-TASSER server included the dsRNA binding proteins, 1YYK, 2L3J, and 1DI2. Most of these structures were resolved through NMR or X-ray diffraction studies in the presence of RNA duplexes or hairpins (synthetic or natural). T-COFFEE version 11.0 was used for pairwise alignment of 1YYK (template) and the p4a sequence. Initially, Modeller^42, 43^ was used to build the protein model. Also, four other online tools including I-TASSER^44^, PHYRE^45^, Swiss model^46^, and CPH model^47^ were used for comparative analysis and validation of the protein models. The best model was selected based on the minimum violation of atomic contacts and maximum score criteria. The quality and accuracy of the built model was further evaluated and validated using a Ramachandran plot^48^, QMEAN^49^, and ProSA^50^. Energy minimization was performed for model refinement and quality improvement using UCSF Chimera^51^. To add charges on standard amino acids, AMBER ff99SB force field was used while AM1BCC force field was applied to other residues. The steepest descent minimization steps were set to 100, whereas the conjugate gradient was set to 10 with a step size of 0.02 Å. The secondary structure of the modeled protein was validated using Protein Structure Validation Suite (PSVS)^52^ and Protein Structure Evaluation Suite and Server (ProSESS)^53^. The secondary structure elements of the 3D model were compared with those of the predicted ones in order to measure the structural reliability. The selected p4a model was subjected to MD simulations to obtain a fully optimized and stable structure for further analysis (further discussed in the molecular dynamics section).

To get the desired p4a-dsRNA complex, a 21-base pair long hairpin (dsRNA)^54^ was selected from PDB and docked with the fully optimized p4a using the HEX docking server^55^. While predicting the functional annotation of the query protein, the same dsRNA structure (reported in PDB structure 2LK2) was suggested by I-TASSER, among the other top three suggested structures (1YYO, 2LK2, and 1DI2). The HEX docking method is based on the rigid body docking algorithm that explicitly considers the electrostatic potential, steric shape, and charge density of the protein^55^. Parameters used for docking were set to default except that the correlation type was set to shape and electrostatics. Intermolecular interactions were checked using UCSF Chimera^51^.

### Alanine Scanning Mutagenesis

*In silico* mutagenesis was performed using the MOE that calculates the dAffinity for a particular amino acid residue. The detailed mechanism of this alanine scanning mutagenesis approach has been discussed previously^56^. The MOE program also calculates dStability of a mutant complex with respect to a wild type amino acid residue. I-Mutant server^57^ was also used to predict the changes in protein stability due to the mutations. It is a support vector machine-based server, which predicts the stability of a given protein after point mutations in terms of ΔΔG at the following physiological conditions: pH of 7.0 and temperature of 25 °C.

### Molecular Dynamic Simulations

The modeled 4a protein, p4a-dsRNA wild type complex, and p4a-dsRNA mutant complexes, suggested by the experimental mutational analysis and our *in silico* alanine scanning, were subjected to MD simulations. Six systems, including p4a-dsRNA wild type and mutants N8A, K27A, W45A, K63A, and K67A, were prepared and investigated for structural dynamics. Each system was simulated for 200 ns using Gromacs 5.0^58^ with the Amber99SB-ILDN force field^59^. Periodic boundary conditions were applied to mimic the infinite system with an octahedron box by keeping a 10 Å distance between the protein’s surface and box boundary. The total charge of each system was neutralized by adding counter ions at the physiological concentration of 0.15 M salt. The long-range electrostatic interactions were computed using the particle mesh Ewald algorithm^60^. The LINCS algorithm^61^ was applied to constrain bond lengths. The systems were energy-minimized using the steepest descent algorithm to remove steric clashes between atoms. The energy-minimized systems were simulated with NVT ensemble for 100 ps followed by NPT ensemble for 100 ps in order to equilibrate temperature and pressure, respectively. The temperature and pressure were coupled with V-rescale^62^ and Parrinello-Rahman barostat methods^62^, respectively. The production run was carried out for 200 ns without backbone restraints with a time step of 0.002 ns under the NPT condition. The trajectory data was visualized and analyzed through PyMOL^63^, Chimera, and MOE. All plots were drawn using the XMgrace program [http://plasma-gate.weizmann.ac.il/Grace/].

### Essential dynamics and Gibbs energy calculation

The trajectory files obtained from MD simulations were used to explore the dominant motions in wild type and mutant complexes through PCA or essential dynamics^64, 65^. The rotational and translational motions of the coordinates were eliminated and subsequently superimposed onto a reference structure. Afterward, we calculated the positional covariance matrix of atomic coordinates and its eigenvectors. The matrix was diagonalized by an orthogonal coordinate transformation matrix yielding the diagonal matrix of eigenvalues. The first eigenvector and its corresponding eigenvalue usually indicate the principal component of the trajectory, which contains the principal dominant global motion of the structures. The extent and direction of the most dominant motions of all complexes were visualized through porcupine plots using the ‘modevectors.py’ script (written by Sean M. Law) in PyMOL.

FELs were obtained by plotting the first two principal components (PC1 and PC2) against each other, obtained from the PCA of the last 100 ns MD trajectories of each system. The corresponding Gibbs energy represents conformations of molecules obtained through the trajectory. The deep valleys represent stable and dominant conformations and boundaries represent intermediate conformations of the molecules^66^. The g_sham function distributed in GROMACS was used for the Gibbs energy calculations and the trial version of Mathematica^67^ was used to obtain 3D images of the plots. Coordinates on 3D images were used to find exact time frames and snapshots of molecules at a particular time and state.

The DCCM was constructed to identify correlated motions of residues. The matrix (*C*_*ij*_) depicts the time-correlated information between the *i* and *j* atoms of a protein^68, 69^. To construct the matrix, only Cα atoms from the last 500 snapshots were selected at 0.002 ns time intervals. The positive values indicate motions in the same direction or correlated motions whereas negative values mean atomic displacement in the opposite direction.

### Binding Free Energy Calculations

The MMPBSA method was used to calculate the free energy of binding between wild type and mutant complexes. A total of 500 conformations extracted at 0.2 ns time intervals from the last 10 ns trajectories were subjected to calculation using the ‘g_mmpbsa’ tool^70^. A detailed description of methods used in the calculation can be found in previous studies^71–73^.

## Electronic supplementary material

Supplementary Information

PDB files
