# Supplementary material for: Structural insights into the Middle East respiratory syndrome coronavirus 4a protein and its dsRNA binding mechanism
Source: Sci Rep. 2017 Sep 12;7:11362. doi: 10.1038/s41598-017-11736-6 (PMC5596018; doi:10.1038/s41598-017-11736-6)
Supplement: Supplementary file 1 — Supplementary Information [file 41598_2017_11736_MOESM1_ESM.pdf]

<Supplementary Information>

Structural insights into the Middle East respiratory syndrome coronavirus 4a protein and its dsRNA binding mechanism

Maria Batool, Masaud Shah, Mahesh Chandra Patra, Dhanusha Yesudhas, and Sangdun Choi*

Department of Molecular Science and Technology, Ajou University, Suwon, 16499, South Korea

***Corresponding Author**

Sangdun Choi, PhD

Department of Molecular Science and Technology

Ajou University, Suwon, 16499, Korea

Phone: +82-31-219-2600

Fax: +82-31-219-1615

E-mail: sangdunchoi@ajou.ac.kr

Supplementary Table S1 | Comparative validation of p4a 3D models. This table summarizes the validation scores of p4a models obtained from different servers/tools. Ramachandran plot values were obtained from RAMPAGE server, ERRAT quality factor values were obtained from NIH server, Z-scores were obtained from ProSA-web, and QMEAN scores were obtained from QMEAN server. The model obtained from I-TASSER showed the best results among all.

Modelling Servers	No. of residues	Ramachandran Plot values (%)	ERRAT Quality Factor	*Z-score	**QMEAN
**I-TASSER**	72	Favored region: 90<br>Allowed region: 9<br>Outliers: 1	81.967	-2.9	-2.45
**Modeller**	74	Favored region: 87.5<br>Allowed region: 6.9<br>Outliers: 5.6	71.212	-1.78	-3.83
**CPHmodels**	70	Favored region: 83.8<br>Allowed region: 13.2<br>Outliers: 2.9	67.742	-1.62	-3.35
**Swiss-Model**	71	Favored region: 78.3<br>Allowed region: 15.9<br>Outliers: 5.8	80.328	-2.06	-6.16
**PHYRE**	61	Favored region: 86.4<br>Allowed region: 6.8<br>Outliers: 6.8	67.925	-1.85	-3.34

**Z-score: Z-score indicates the overall model quality using C-alpha atoms*
*** QMEAN: Qualitative Model Energy Analysis (Models of low quality are expected to have strongly negative QMEAN Z-scores)*

Supplementary Table S2 | Alanine scanning mutagenesis. The amino acid residues present at the p4a-dsRNA interacting interface were selected and mutated into alanine using protein design package distributed in MOE. The relative binding affinities and stabilities (dAffinities and dStabilities) of the mutant to wild-type residues were calculated using LowModeMD ensemble. The LowModeMD Search method generates conformations using ~1 ps run of molecular dynamics (MD) at constant temperature (300 K) followed by an all-atom energy minimization. The more positive dAffinity value represents the importance of wild-type residue at that particular position. Mutations with negative value indicates the relatively less importance of the wild-type residues. The top five mutations include the two experimentally reported mutations, K67A and K63A. Besides the already reported residues, other amino acid residues suggested in the table below (K27A, W45A, and N8A) might also be crucial in p4a-dsRNA interaction. The individual role of these tope five residues has been elaborated in the main text.

Mutation	dAffinity	dStability
**K27A**	10.109	0.147
**W45A**	5.611	5.605
**N8A**	3.359	0.523
**K67A**	0.578	-0.202
**K63A**	0.225	-0.709
**T38A**	-0.716	0.195
**Y3A**	-1.785	1.912
**Q12A**	-1.994	0.733
**S38A**	-3.168	-0.043
**G37A**	-5.048	-2.583

The unit for dAffinity and dStability is kcal/mol

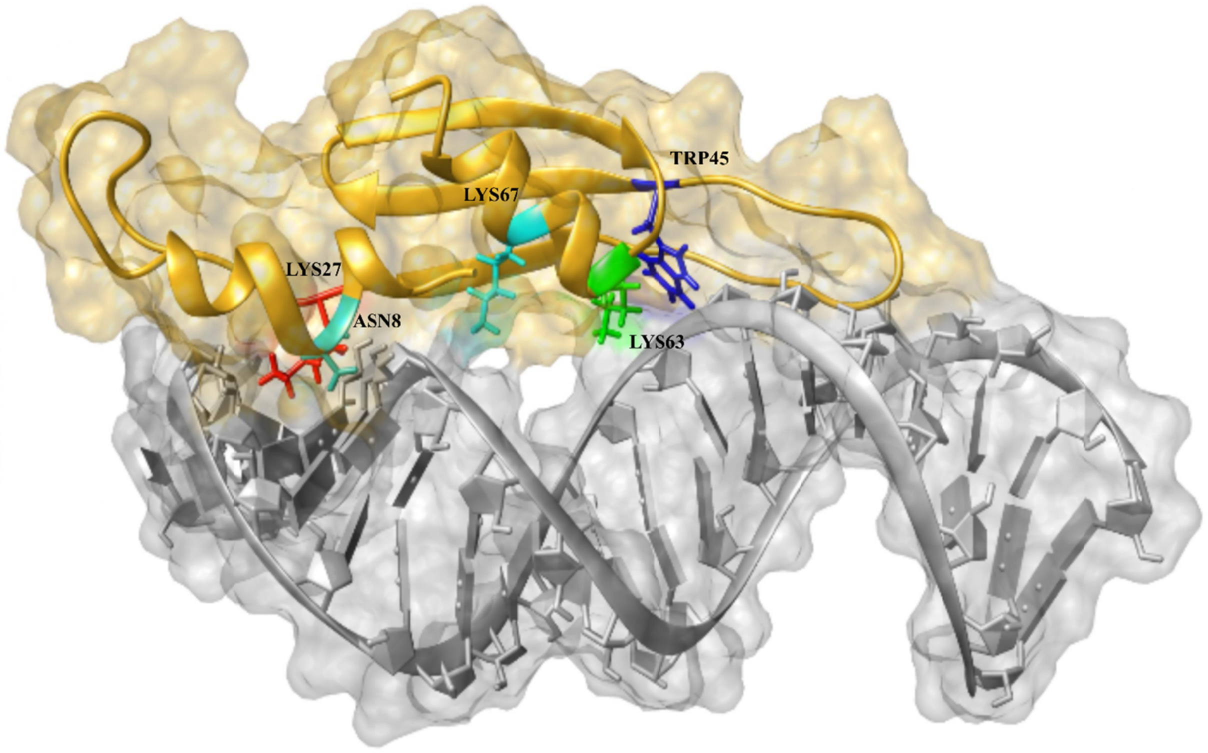

Supplementary Figure S1 | Complex of MERS-CoV p4a and dsRNA. The binding interface of p4a-dsRNA complex obtained from HEX docking server. The hotspot residues N8, K27, W45, K63, and K67 are highlighted. These residues are located in helix α1, helix α2 and loop 2 of p4a and play crucial roles in p4a-dsRNA interaction.

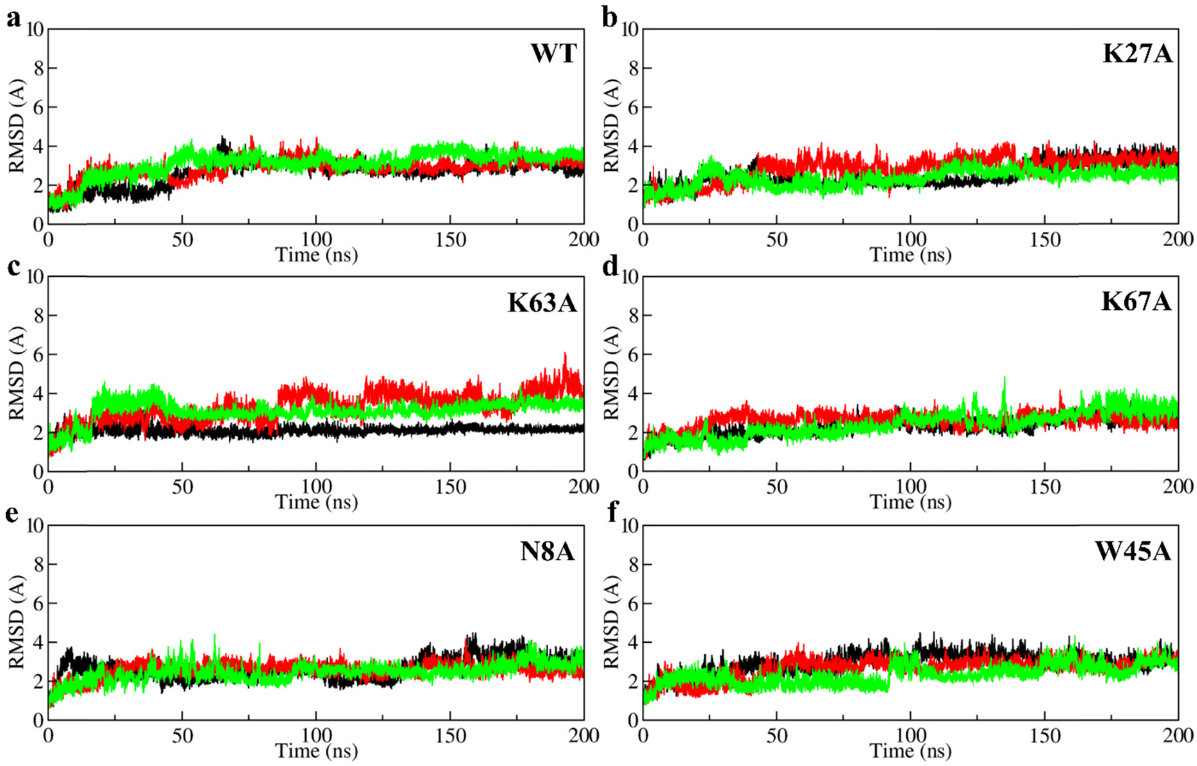

Supplementary Figure S2 | The root mean square deviation (RMSD) of MERS-CoV p4a variants' backbone atoms during three independent MD simulations. Three MD simulations were performed for each system using different initial velocities. Black, red, and green colors represent RMSDs from first, second, and third MD trajectory, respectively. The RMSD plots of each set of p4a variants converged toward the end of simulation, except K63A that exhibited relatively higher backbone deviations after 100 ns.

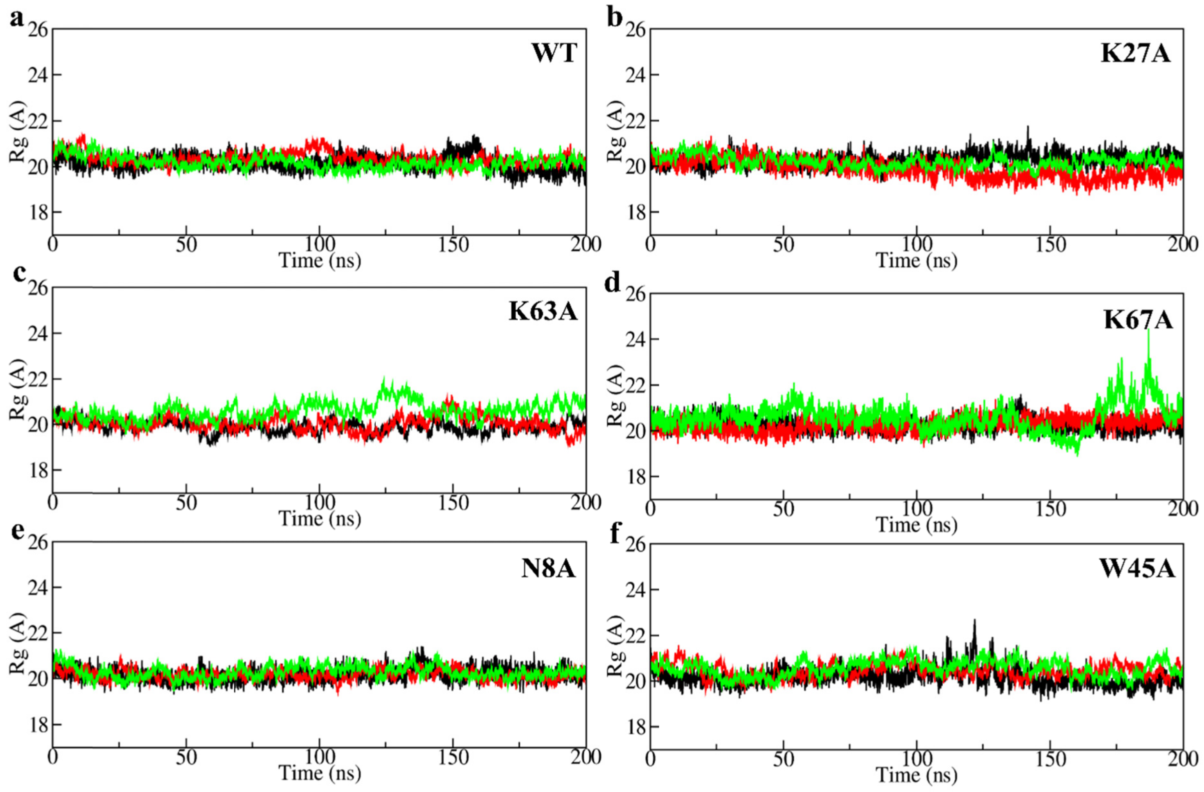

Supplementary Figure S3 | The Radius of gyration (Rg) plots of MERS-CoV p4a variants during three independent MD simulations. Black, red, and green colors represent Rg from first, second, and third MD trajectory, respectively. Rg of all MERS-CoV p4a-dsRNA complexes oscillated around 20 Å during MD simulation.

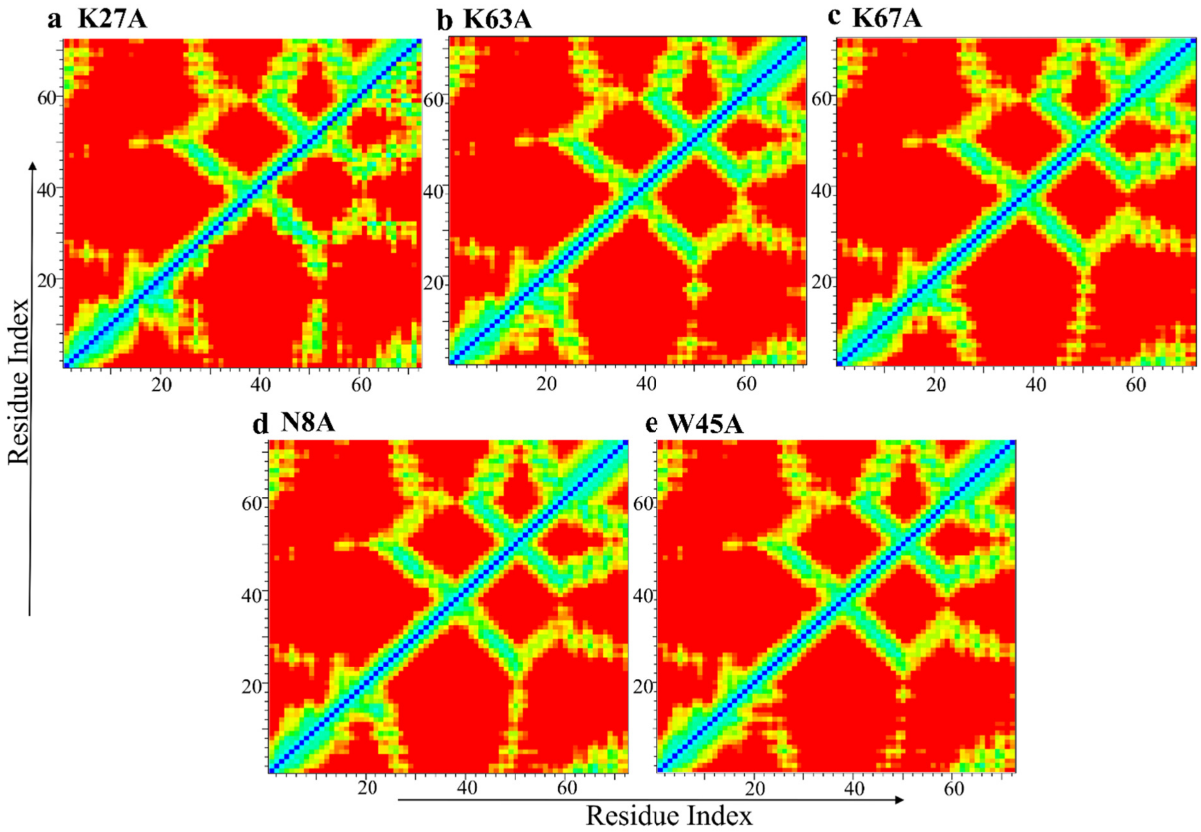

Supplementary Figure S4 | Residue contact map of p4a protein. The contact map of wild-type and mutant residues highlights the movements at the residue level. The upper half of each correlation plot represents the contact map of wild-type protein, whereas the lower half represents the mutant complex. Comparatively, K27A mutant exhibited a distinctive contact map; this suggests that K27 could be a crucial for p4a-dsRNA complex stability.

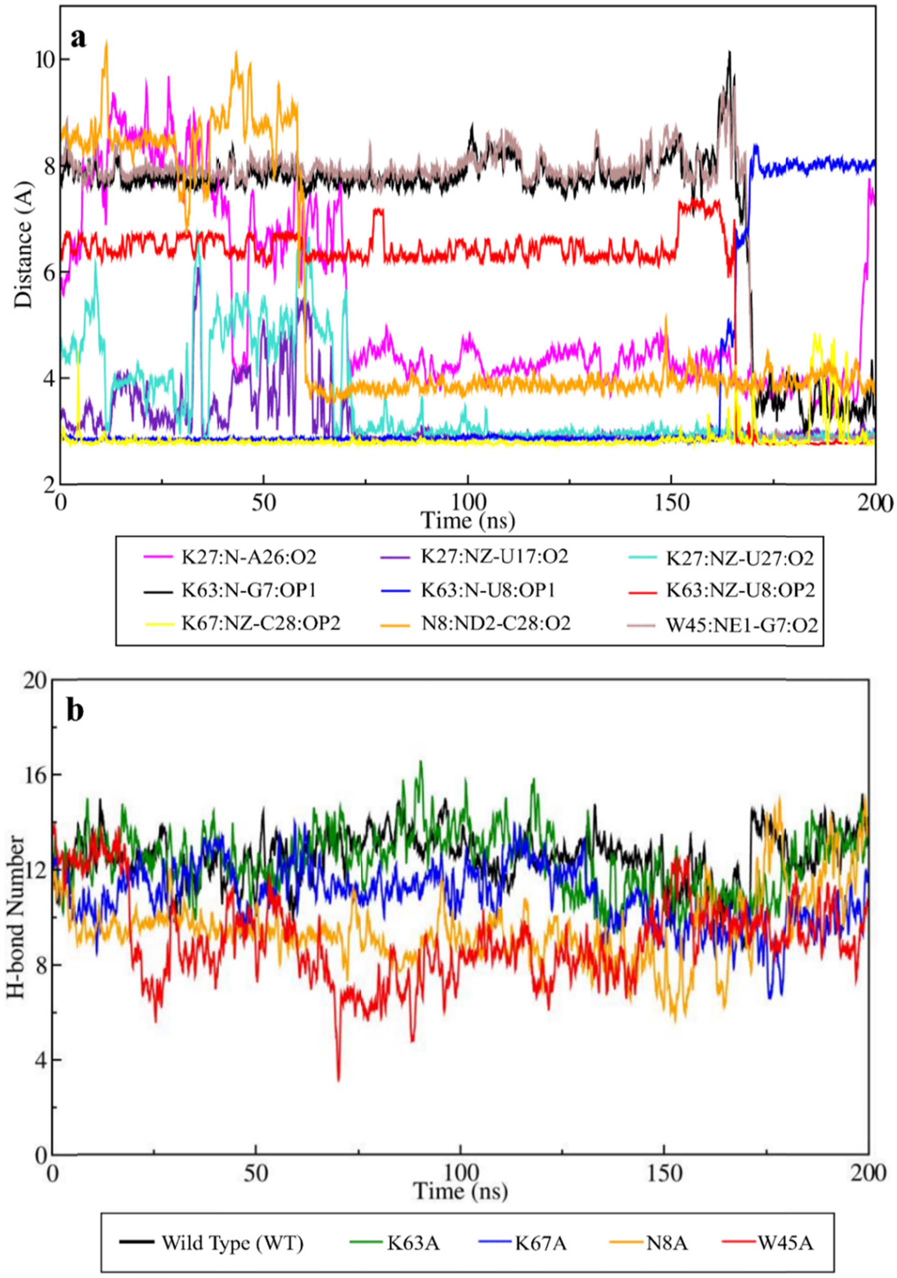

Supplementary Figure S5 | Hydrogen bond (h-bond) analyses of p4a-dsRNA complex as a function of time. (a) Variation in h-bond distances between wild-type p4a's hotspot residues and dsRNA nucleotides. (b) Change in the total number of h-bonds between p4a and dsRNA during MD simulation. A decrease in the number of h-bonds between p4a^{W45A} mutant and dsRNA was observed. The corresponding color codes have been provided at the bottom of each figure.

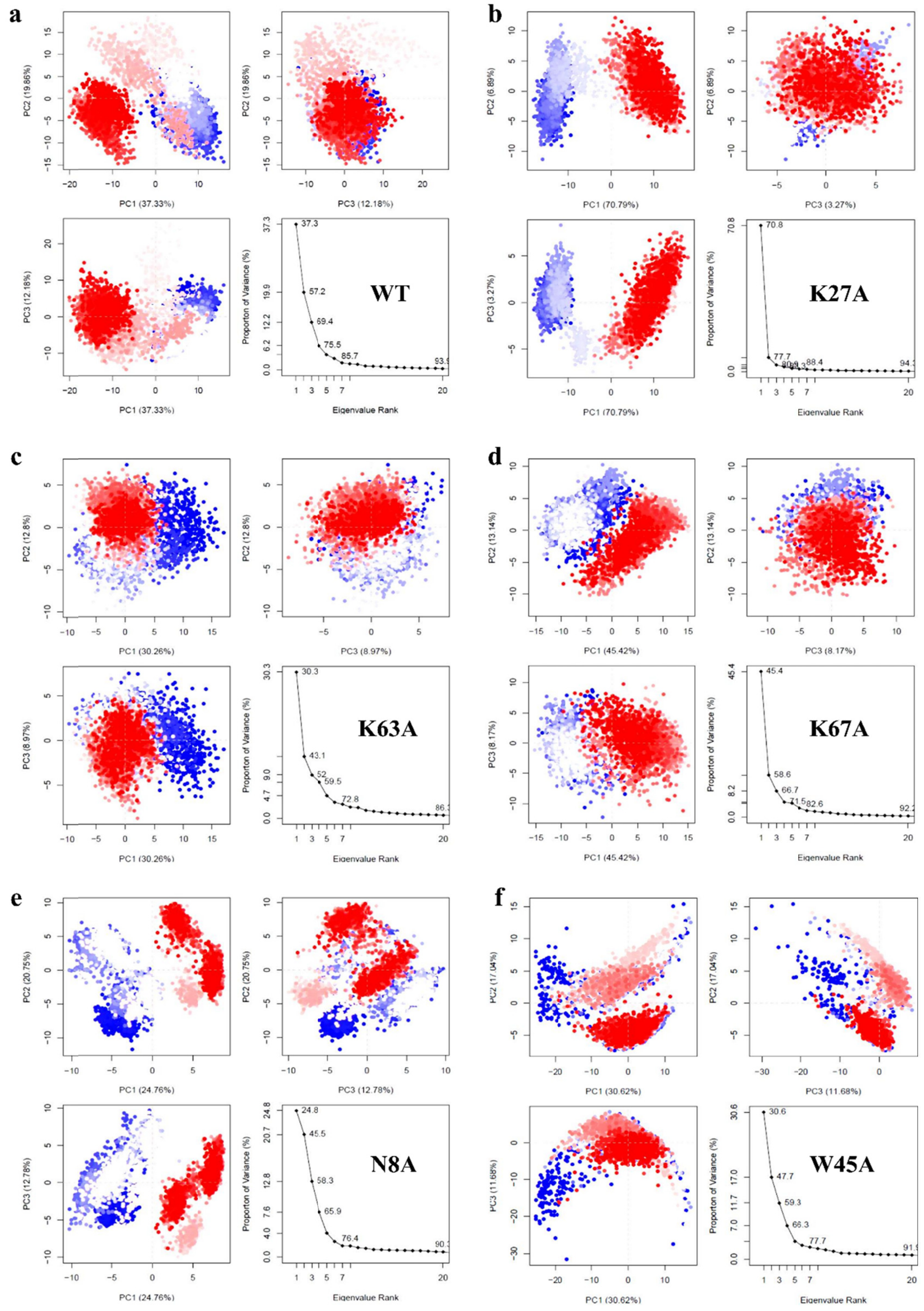

Supplementary Figure S6 | Principal component analysis (PCA) of p4a variants. 2D plots

represent the projection of motions of p4a and its variants by plotting the first three eigenvectors obtained from the last 100 ns trajectories. Color scale tracks the movement of eigenvectors during the trajectory from blue to red. The percentage of principal motions represented by their corresponding eigenvectors are shown in the lower half of each panel. ~80% of the motions correspond to the first 5-6 eigenvectors in wild type as well as mutant p4a.

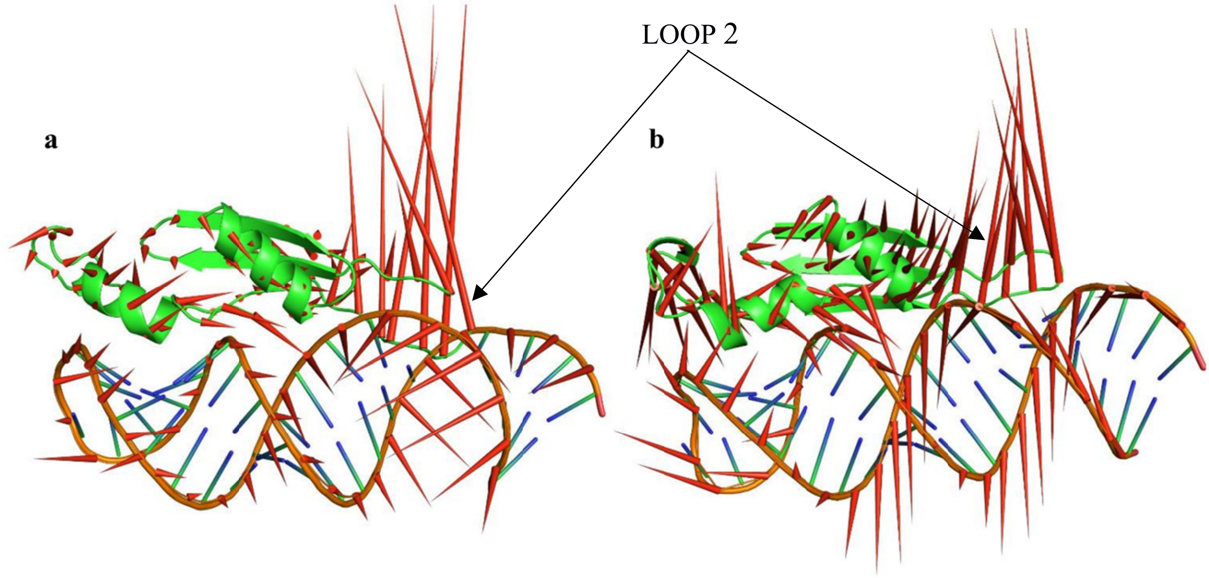

Supplementary Figure S7 | Porcupine plots of K27A and W45A complexes. Porcupine plot represents amplitude and direction of the most dominant motions in p4a and bound dsRNA. The direction and magnitude of movements of loop 2 and dsRNA can be clearly seen from the angle and lengths of the spikes, respectively. (a) In K27A, dsRNA moves in opposite direction of the loop 2. (b) In W45A, loop 2 exhibits a more prominent movement in the opposing direction of dsRNA. However, helix α1 and helix α2 tend to move toward dsRNA. This suggests that dsRNA does not move in concert with the fluctuating loop 2.
